# Predictive value of blood composite biomarkers for hemorrhagic transformation following mechanical thrombectomy in acute anterior circulation large vessel occlusion stroke patients

**DOI:** 10.3389/fnins.2026.1907532

**Published:** 2026-09-14

**Authors:** Mujie Yao, Jun Li, Sen Xu, Yue Wan

**Affiliations:** 1 School of Medicine, Wuhan University of Science and Technology, Wuhan, China; 2 Department of Neurology, Hubei No. 3 People’s Hospital of Jianghan University, Wuhan, China

**Keywords:** acute ischemic stroke, composite biomarkers, glucose-to-lymphocyte ratio, hemorrhagic transformation, mechanical thrombectomy, predictive model

## Abstract

**Background:**

Hemorrhagic transformation (HT) is a devastating complication of mechanical thrombectomy (MT) for acute anterior circulation large vessel occlusion (LVO), yet reliable early prediction tools remain limited. This study systematically compared nine blood composite biomarkers to identify the optimal metabolic-immune integrative predictor of HT.

**Methods:**

A total of 206 patients with acute anterior circulation LVO who underwent MT were retrospectively enrolled. Nine composite biomarkers were calculated from routine admission laboratory tests: platelet-to-lymphocyte ratio (PLR), neutrophil-to-platelet ratio (NPR), systemic inflammation response index (SIRI), pan-immune inflammation value (PIV), monocyte-to-HDL ratio (MHR), neutrophil-to-lymphocyte ratio (NLR), systemic immune-inflammation index (SII), glucose-to-potassium ratio (GPR), and glucose-to-lymphocyte ratio (GLR). A stratified screening strategy was employed: elastic net regression for baseline variable selection, Spearman correlation for biomarker clustering, and univariate AUC with DeLong test for representative selection. Model evaluation incorporated discrimination (AUC with DeLong test and Bootstrap 1,000-iteration optimism correction), calibration (Hosmer-Lemeshow test and Brier score), and clinical net benefit (NRI, IDI, and decision curve analysis).

**Results:**

HT occurred in 53 patients (25.7%). Elastic net regression identified six baseline variables: mean platelet volume, blood glucose, D-dimer, history of alcohol consumption, leukoaraiosis, and pulmonary infection. Spearman clustering yielded five representative biomarkers: GLR, SIRI, GPR, NPR, and MHR. Among these, GLR was the only biomarker that showed a trend toward improving? the baseline model’s discrimination (AUC: 0.852–0.882; △AUC = 0.030), although this difference did not reach statistical significance (*P* = 0.057). Bootstrap-corrected AUC for Base+GLR was 0.861. GLR also showed the most substantial calibration improvement (Hosmer-Lemeshow *P* = 0.858 vs. 0.081 for baseline; Brier score reduction 5.4%) and achieved significant integrated discrimination improvement (IDI = 0.0376, 95% CI: 0.002–0.078) and continuous net reclassification improvement (NRI = 0.712). Decision curve analysis revealed limited net benefit difference between GLR and the baseline model, suggesting GLR requires integration with multidimensional factors for comprehensive assessment.

**Conclusion:**

Among nine composite biomarkers, GLR provides the greatest incremental predictive value for HT following MT in acute anterior circulation LVO patients. As a routinely accessible metabolic-immune integrative index, GLR may serve as an early warning layer for perioperative risk stratification, though integration with imaging and procedural information remains essential for clinical decision-making.

## 1 Introduction

Acute ischemic stroke (AIS) represents a major global health concern characterized by high disability and mortality rates. Stroke resulting from large vessel occlusion (LVO) comprises approximately 24–46% of AIS cases (Lakomkin et al., 2018; Rennert et al., 2019). Recent advancements in endovascular therapy, particularly mechanical thrombectomy (MT), have demonstrated substantial progress. Multiple randomized controlled trials and meta-analyses indicate that MT combined with standard medical treatment significantly improves functional outcomes in patients with anterior circulation LVO (Goyal et al., 2016).

Mechanical thrombectomy has become the standard treatment for acute anterior circulation LVO ischemic stroke. However, post-MT complications, such as hemorrhagic transformation (HT), significantly impair neurological recovery and clinical outcomes. The incidence of HT post-MT varies considerably among cohorts, with reported intracranial hemorrhage rates ranging from 16 to 46% (Luff et al., 2025). Hemorrhagic transformation is established as an independent predictor of poor prognosis regardless of clinical symptom manifestation (Jiang et al., 2024; Yuan et al., 2021). Thus, systematic elucidation of hemorrhagic transformation mechanisms post-mechanical thrombectomy and development of reliable early prediction tools are crucial for optimizing perioperative management and improving clinical outcomes.

In prior research using a cohort of acute anterior circulation LVO patients undergoing mechanical thrombectomy, our group developed a nomogram model incorporating history of alcohol consumption, leukoaraiosis, GLR, D-dimer, blood glucose, and mean platelet volume, which demonstrated strong discrimination (AUC = 0.880) and calibration (Hosmer-Lemeshow test *P* = 0.337) (Yao et al., 2026). Among these variables, GLR as a composite biomarker specifically drew our attention. Compared to single inflammatory biomarkers, composite biomarkers provide a more precise reflection of systemic inflammatory status and prognosis, rendering them valuable for clinical prognostication in critical care settings. Nevertheless, the optimal blood-based composite biomarker for predicting HT after MT remains undetermined.

The present study extends this prior work by moving from single-biomarker validation to systematic multi-marker comparison. Unlike our previous analysis that pre-specified GLR into a nomogram (Yao et al., 2026), this investigation addresses a broader clinical question: among all available composite biomarkers, which provides the greatest incremental predictive value beyond traditional clinical-laboratory variables? Consequently, this study aims to investigate the predictive value of several novel blood composite biomarkers for HT following MT in acute anterior circulation LVO stroke patients, employing a rigorous statistical framework encompassing elastic net regression, DeLong testing, Bootstrap optimism correction, and net reclassification indices, hoping to establish an accurate and readily accessible assessment method for HT prediction.

## 2 Subjects and methods

### 2.1 Subjects

A total of 206 patients with acute anterior circulation LVO who underwent mechanical thrombectomy in the Neurology Department of Hubei Third People’s Hospital were retrospectively and consecutively enrolled from January 2023 to March 2026. The study protocol was approved by the Ethics Committee of Hubei No. 3 People’s Hospital of Jianghan University (Approval No. HBZS 2026-C013-01). All patients were anonymized.

A subset of patients in this cohort (*n* = 193) was included in our previous study (Yao et al., 2026), which developed a baseline nomogram incorporating GLR for HT prediction. The present study represents a distinct analytical framework: rather than validating a single pre-specified biomarker, we conducted a systematic comparative evaluation of nine composite biomarkers using elastic net regression, DeLong testing, and NRI/IDI quantification to identify the optimal metabolic-immune integrative index.

Inclusion criteria: (1) Compliance with acute ischemic stroke diagnostic criteria and meeting indications for mechanical thrombectomy (Chinese Society of Neurology et al., 2022); (2) Age greater than 18 years; (3) Radiologically confirmed anterior circulation LVO eligible for thrombectomy; (4) Complete clinical data available for the variables included in the final analysis.

Exclusion criteria: (1) Preoperative intracranial hemorrhage; (2) Concurrent malignancy or severe organ failure; (3) Severe organic diseases in vital organs (heart, liver, kidney) or systemic severe infectious diseases; (4) History of bleeding (gastrointestinal or urinary tract) within the past month; (5) Severe infection; (6) Intracranial aneurysm or arteriovenous malformation; (7) Brain tumors exhibiting mass effect on imaging.

### 2.2 Grouping criteria and group allocation

According to the Chinese Consensus on Diagnosis and Treatment of Hemorrhagic Transformation in Acute Cerebral Infarction (Chinese Society of Neurology et al., 2019), HT is defined as the condition where no intracranial hemorrhage is detected by cranial computed tomography (CT) or magnetic resonance imaging (MRI) prior to MT, but re-imaging within 24–72 hours postoperatively reveals hemorrhage within the infarct territory or at remote sites (Leigh et al., 2024). Dynamic cranial CT scans were conducted on all patients within 24–72 h post-procedure. Patients exhibiting parenchymal hemorrhage, intraventricular hemorrhage, or subarachnoid hemorrhage (Romanic et al., 1998) were classified into the HT group; all others were assigned to the Non-HT Group.

Due to the retrospective nature of this study and limitations in electronic medical record documentation, HT subtyping according to the ECASS classification (HI1/HI2/PH1/PH2) or differentiation between symptomatic and asymptomatic HT was not consistently available. This represents a limitation of the current analysis.

All patients underwent mechanical thrombectomy under local anesthesia in the supine position. Following standard skin disinfection and draping, femoral artery access was established via Seldinger technique with placement of an 8F arterial sheath, followed by comprehensive cerebral angiography to delineate the occlusive lesion location. A 6F guiding catheter was positioned proximal to the occlusion site, whereupon an intermediate catheter was advanced into the target vessel. A microguidewire was then used to navigate a microcatheter and stent retriever to the thrombus site. Post-deployment contrast injection confirmed accurate positioning and complete expansion. After waiting 5 min, the stent and catheter were simultaneously withdrawn while maintaining continuous negative pressure aspiration. Repeat angiography was then performed to evaluate vascular recanalization, with additional thrombectomy procedures conducted as clinically indicated. Tirofiban was administered via intracatheter injection in cases of vessel re-occlusion or residual thrombus. Final angiography was performed approximately 30 min later, and the procedure was terminated upon confirmation of vascular patency.

All 206 AIS patients successfully underwent mechanical thrombectomy, with 53 developing hemorrhagic transformation (HT group) and 153 without HT (Non-HT group).

### 2.3 Data collections

#### 2.3.1 Basic information

Patient information was extracted from electronic medical records, including:

(1) Baseline characteristics: age and sex; history of alcohol consumption and smoking, along with histories of diabetes, hypertension, atrial fibrillation, and cerebral infarction;
(2) Laboratory indices: neutrophil count, lymphocyte count, monocyte count, platelet count, mean platelet volume, platelet distribution width, uric acid, serum potassium, glomerular filtration rate (GFR), serum glucose, triglycerides, high-density lipoprotein (HDL), low-density lipoprotein (LDL), C-reactive protein, fibrinogen, activated partial thromboplastin time (APTT), and D-dimer;
(3) Procedure-related variables: access vessel, procedure duration, administration of intra-arterial thrombolysis;
(4) Other relevant variables: admission National Institutes of Health Stroke Scale (NIHSS) score, culprit vessel, preoperative intravenous thrombolysis administration, preoperative antiplatelet use, preoperative anticoagulant use, preoperative pulmonary infection.

#### 2.3.2 Composite biomarker calculation methods

Peripheral venous blood samples were collected from all patients within 24 h of admission. With the exception of lipid profiles, all parameters were measured preoperatively. Laboratory parameters were obtained via the LIS system and used to calculate blood composite biomarkers. Composite biomarker components and computational methods are detailed in Table 1.

**TABLE 1:** Composite biomarker calculation method.

Item	Calculation method
PLR	Platelet count /lymphocyte count
NPR	Neutrophils count/platelet count
SIRI	Neutrophils count × monocytes count/lymphocyte count
PIV	Neutrophils count × monocytes count × platelet count/lymphocyte count
MHR	Monocytes count/HDL-C (mmol/L)
NLR	Neutrophils count/lymphocyte count
SII	Platelet count × neutrophils count/lymphocyte count
GPR	Blood glucose (mmol/L)/serum potassium (mmol/L)
GLR	Blood glucose (mmol/L)/lymphocyte count

## 3 Statistical analysis methods

Statistical analysis was performed using R 4.5.1 software. The flow chart is shown in Figure 1.

**FIGURE 1:**
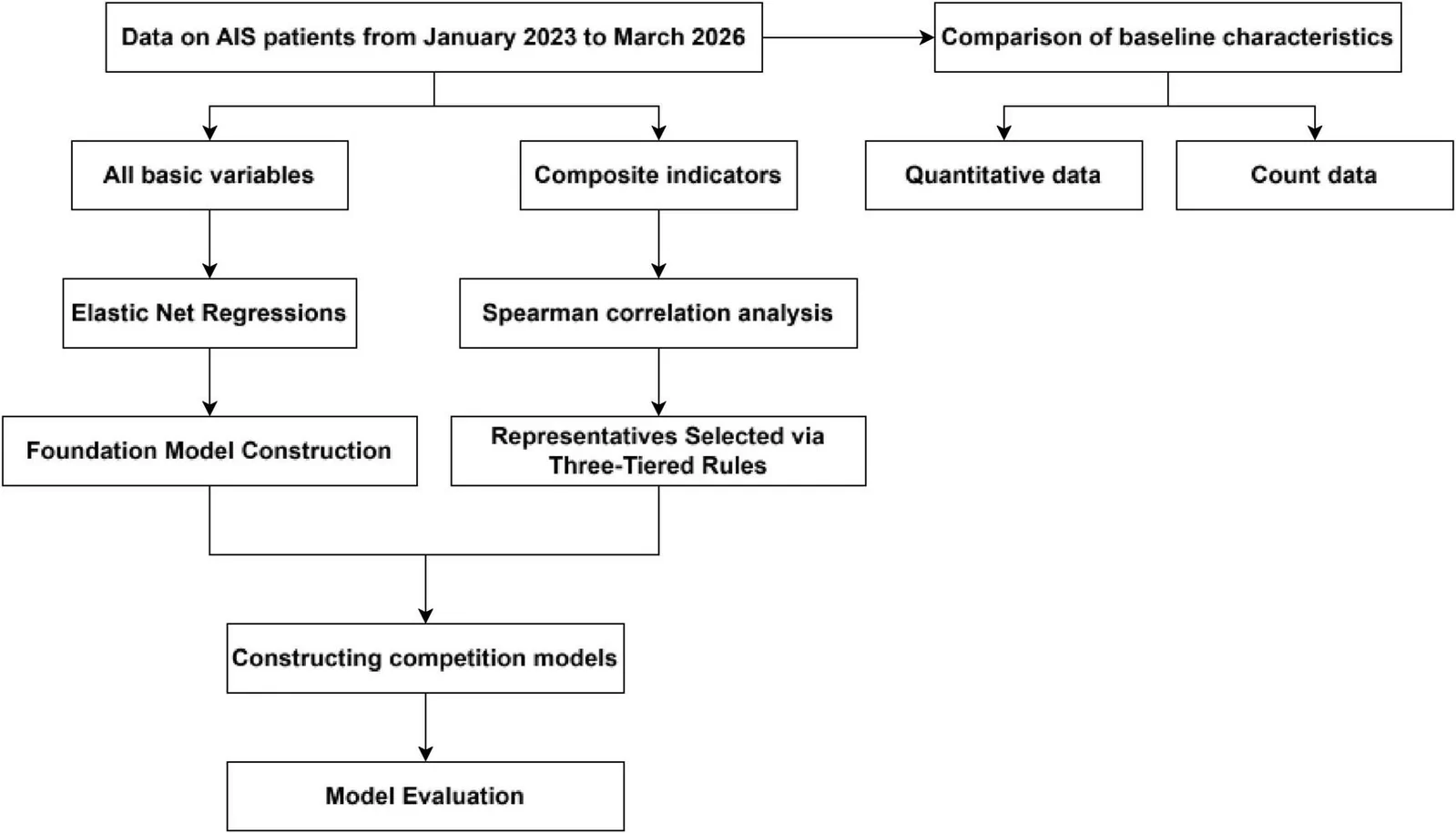
Statistical analysis flow chart.

### 3.1 Univariate analysis of factors

Normality of measurement data was assessed using the Shapiro-Wilk test. Normally distributed measurement data are expressed as mean ± standard deviation (x̄ ± s), and group comparisons were performed using independent samples *t*-tests; non-normally distributed measurement data are expressed as median (interquartile range) [M(P25, P75)], and group comparisons were performed using the Mann-Whitney U test. Categorical data are expressed as frequency (percentage), and group comparisons were performed using χ^2^ tests or Fisher’s exact test.

### 3.2 Construction of the competition model

Given the functional dependency between composite inflammatory biomarkers and baseline hematological variables, coupled with high correlations among various composite biomarkers, this study employed a stratified screening strategy:

First, using all baseline variables as the candidate set, elastic net regression was applied for variable selection (α range: 0.1–0.9 via Grid Search, optimal λ determined through 10-fold cross-validation using the λ.1se criterion). Significant biomarkers were subsequently incorporated into binary logistic regression to construct the baseline model.

Second, Spearman correlation analysis was conducted on the nine composite biomarkers; biomarkers with | *r*| > 0.80 were grouped into clusters, while those with | *r*| ≤ 0.80 were classified as independent indicators. Within each biomarker family, representative selection followed three hierarchical rules: Rule 1 prioritized the marker with the highest univariate AUC; if the AUC difference between the top two candidates was ≥ 0.01, significance was confirmed using the DeLong test (*P* < 0.05). Rule 2 selected the marker with broader clinical validation if Rule 1 could not determine; Rule 3 chose the marker with fewer composite variables if still undifferentiated. The ultimately retained representative biomarkers were combined with the baseline model to construct competitive models.

### 3.3 Model evaluation

Model evaluation was conducted across three dimensions:

(1) Discrimination ability: measured by the area under the receiver operating characteristic curve (AUC), with DeLong test comparing AUC between competitive and baseline models;
(2) Calibration: assessed using the Hosmer-Lemeshow goodness-of-fit test (*P* > 0.05 indicates good calibration) and the Brier score;
(3) Clinical Net Benefit: incremental predictive value quantified via the Net Reclassification Improvement index (NRI, including both event and non-event groups) and Integrated Discrimination Improvement index (IDI). Standardized net benefit at different threshold probabilities was evaluated using Decision Curve Analysis (DCA).

Internal validation employed the Bootstrap method (1,000 repetitions) to compute the corrected AUC and evaluate model optimism. The optimism was calculated as the difference between the original (apparent) AUC and the mean Bootstrap AUC: Optimism = mean (AUC_bootstrap) - AUC_original. The corrected AUC was derived as: AUC_corrected = AUC_original - Optimism.

Statistical significance was defined as *P* < 0.05 (two-tailed). Variables with *P*-values between 0.05 and 0.10 were interpreted as marginally significant and reported as trend-level findings.

## 4 Results

### 4.1 Comparison of general characteristics between the two groups

This study enrolled 206 individuals, comprising 153 (74.27%) in the Non-HT group and 53 (25.73%) in the HT group. Statistically significant differences (*P* < 0.05) were observed between the two groups concerning leukoaraiosis history, preoperative pulmonary infection, absolute neutrophil count, absolute lymphocyte count, MPV, and blood glucose distribution. No significant differences were found in the other distributions (Table 2).

**TABLE 2:** Comparison of basic information.

Item	HT group (*n* = 53)	Non-HT group (*n* = 153)	χ ^2^/z/t	*p*
Sex (male/female)	17/36	57/96	0.261	0.609
Smoking history	24(45.3%)	51(33.3%)	1.939	0.164
History of alcohol consumption	21(39.6%)	26(17.0%)	10.198	0.001
Hypertension	30(56.6%)	105(68.6%)	2.015	0.156
Atrial fibrillation	20(37.7%)	45(29.4%)	0.907	0.341
Cerebral ischemia	8(15.1%)	27(17.6%)	0.046	0.830
Diabetes mellitus	13(24.5%)	31(20.3%)	0.21	0.646
Anticoagulant use	10(18.9%)	24(15.7%)	0.104	0.747
Antiplatelet medication use	11(20.8%)	19(12.4%)	1.58	0.209
Intravenous thrombolysis	14(26.4%)	53(34.6%)	0.868	0.352
Leukoaraiosis	22(41.5%)	23(15.0%)	14.65	< 0.001
Pulmonary infection	24(45.3%)	34(22.2%)	9.24	0.002
Target vessel				
Middle cerebral artery	32(60.4%)	110(71.9%)	1.93	0.165
Internal carotid artery	21(39.6%)	43(28.1%)
Age [M (P25, P75)]	69.00(62.00, 78.00)	69.00(61.00, 75.00)	0.749	0.454
Admission NIHSS score [x̄ ± s]	16.85 ± 5.88	14.90 ± 6.63	−1.901	0.059
Neutrophils [M (P25, P75)]	7.70(5.12, 9.81)	5.83(4.59, 8.07)	2.282	0.022
Lymphocyte [M(P25, P75)]	0.86(0.58, 1.23)	1.34(0.87, 1.90)	3.353	< 0.001
Monocytes [M(P25, P75)]	0.37(0.25, 0.50)	0.38(0.29, 0.49)	0.428	0.669
Platelet [M(P25, P75)]	191.00(156.00, 221.00)	218.00(164.00, 256.00)	1.884	0.060
MPV[M(P25, P75), fL]	9.60(9.10, 10.40)	10.40(9.50, 11.70)	3.059	0.002
Serum potassium [M(P25, P75), mmol/L]	3.98(3.65, 4.23)	3.99(3.60, 4.27)	0.643	0.520
Blood glucose [M(P25, P75), mmol/L]	9.40(7.67, 12.80)	7.07(6.16, 8.39)	5.5	< 0.001
C-reactive protein [M(P25, P75), mg/L]	8.40(2.70, 25.00)	5.40(2.10, 17.50)	1.731	0.083
Fibrinogen [M(P25, P75), g/L]	2.94(2.49, 3.49)	2.84(2.38, 3.40)	0.904	0.366
APTT[M(P25, P75), s]	29.20(25.60, 38.40)	28.40(25.90, 33.00)	1.033	0.301
PDW[M(P25, P75), fL]	16.10(15.50, 16.50)	16.00(10.60, 16.30)	1.618	0.106
Glomerular filtration rate [M(P25, P75), ml/min/1.73 m^2^]	84.47(65.42, 104.28)	91.56(72.09, 102.58)	0.985	0.324
UA[M(P25, P75), μmol/L]	311.20(234.00, 396.74)	306.58(244.00, 391.00)	0.162	0.871
Triglyceride [M(P25, P75), mmol/L]	0.93(0.65, 1.33)	1.13(0.75, 1.59)	1.401	0.161
HDL-C[M(P25, P75), mmol/L]	1.19(0.89, 1.41)	1.09(0.87, 1.30)	1.481	0.138
LDL-C[x̄ ± s, mmol/L]	2.42 ± 0.77	2.51 ± 0.98	0.597	0.551
D-Dimer [M(P25, P75), μg/mL]	1.79(0.78, 8.01)	1.00(0.47, 2.84)	3.036	0.002

### 4.2 Comparison of composite blood indicators between patient groups

Results demonstrated elevated levels of PLR, NPR, SIRI, PIV, NLR, SII, GPR, and GLR in the HT group compared to the Non-HT group (all *P* < 0.05), whereas MHR showed no statistically significant intergroup difference (*P* = 0.161) (Table 3).

**TABLE 3:** Comparison of blood composite biomarkers.

Item	HT group (*n* = 53)	Non-HT group (*n* = 153)	*z*	*p*
PLR[M(P25, P75)]	16.67(10.00, 24.62)	10.65(6.89, 17.41)	3.655	< 0.001
NPR[M(P25, P75)]	0.04(0.02, 0.06)	0.03(0.02, 0.04)	3.116	0.002
SIRI[M(P25, P75)]	2.34(1.49, 5.06)	1.67(0.92, 2.92)	2.61	0.009
PIV[M(P25, P75)]	481.65(272.55, 814.45)	309.29(199.61, 634.83)	2.078	0.038
MHR[M(P25, P75)]	0.29(0.19, 0.47)	0.37(0.24, 0.52)	1.401	0.161
NLR[M(P25, P75)]	8.60(3.95, 14.65)	4.70(2.66, 8.21)	3.491	< 0.001
SII[M(P25, P75)]	1547.59(815.41, 3028.33)	917.94(580.66, 1636.78)	2.811	0.005
GPR[M(P25, P75)]	2.32(1.94, 3.09)	1.79(1.52, 2.21)	5.088	< 0.001
GLR[M(P25, P75)]	10.35(6.7, 18.18)	5.53(3.47, 9.40)	4.984	< 0.001

### 4.3 Construction of the baseline model using elastic net regression

Elastic Net regularization was employed for variable selection. A Grid Search over α∈ [0.1, 0.9] was performed to determine optimal hyperparameters using 10-fold cross-validation. The lowest cross-validation error (0.9458) was achieved at α = 0.4. Comparing the λ.min criterion (retaining 19 variables) with the λ.1se criterion (retaining 6 variables), the λ.1se standard significantly reduces model complexity while maintaining comparable predictive accuracy. Thus, the λ.1se criterion was ultimately adopted to determine the included variables: Mean Platelet Volume, Blood Glucose, D-dimer, History of Alcohol Consumption, Leukoaraiosis, and Pulmonary Infection (Figure 2).

**FIGURE 2:**
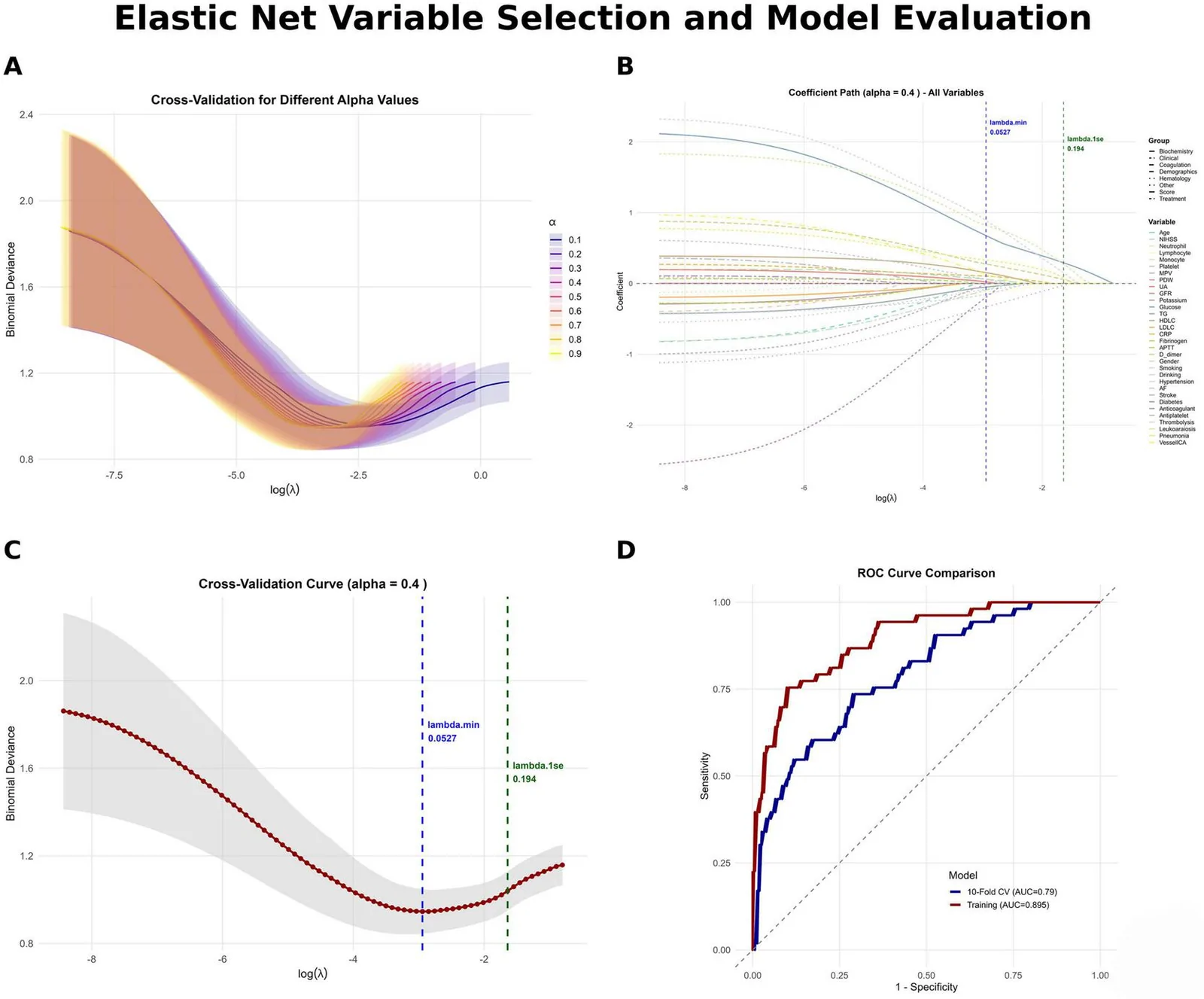
Elastic net variable selection and model evaluation. **(A)** Cross-validated binomial deviance versus log (λ) for α = 0.1–0.9, minimized at α = 0.4. **(B)** Coefficient paths at α = 0.4, with λ.min (0.0527) and λ.1se (0.194) marked by blue and green dashed lines. **(C)** Cross-validation curve at α = 0.4. **(D)** ROC curves in the training set (AUC = 0.895) and under 10-fold cross-validation (AUC = 0.79).

### 4.4 Screening of representative composite biomarkers

Spearman correlation analysis revealed | *r*| = 0.80–0.92 among PLR, NLR, SII, and GLR, composing Family 1; SIRI and PIV showed a correlation of *r* = 0.93, forming Cluster 2; NPR, GPR, and MHR had | *r*| < 0.50 with all other indicators and were identified as independent factors (Figure 3).

**FIGURE 3:**
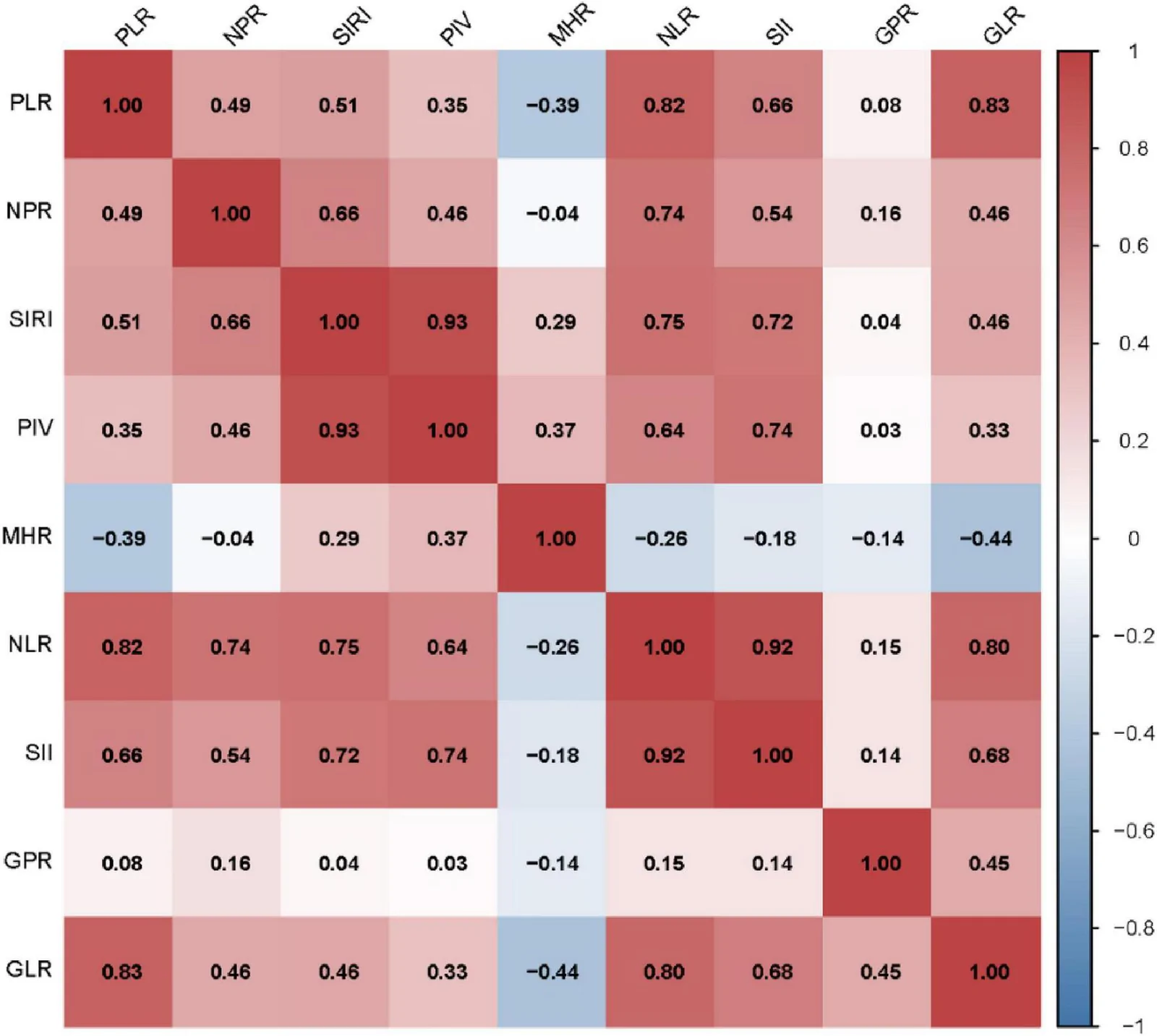
Spearman correlation analysis matrix.

Cluster 1: Univariate analysis revealed GLR had the highest AUC (0.729), exceeding the suboptimal marker SII (AUC = 0.630) by a difference of 0.099 ( ≥ 0.01). The DeLong test confirmed GLR’s AUC was significantly greater than those of PLR (*P* = 0.022), NLR (*P* = 0.019), and SII (*P* = 0.003). Following Rule 1, GLR was retained while PLR, NLR, and SII were eliminated from consideration (Tables 4, 5).

**TABLE 4:** AUC calculation of blood composite biomarkers.

Family	Member	Univariate AUC
Family 1	GLR	0.729
	PLR	0.669
	NLR	0.661
	SII	0.63
Family 2	SIRI	0.62
	PIV	0.586
Independent predictor	GPR	0.735
	NPR	0.644
	MHR	0.565

**TABLE 5:** DeLong test for predictive efficacy of composite biomarkers.

Comparison	Δ AUC	95% CI	*Z*-value	*P*-value
GLR vs. PLR	0.061	0.009∼0.114	2.287	0.022
GLR vs. NLR	0.069	0.011∼0.127	2.341	0.019
GLR vs. SII	0.1	0.034∼0.167	2.957	0.003
SIRI vs. PIV	0.025	−0.008∼0.057	1.499	0.134

Cluster 2: The AUCs for SIRI and PIV were 0.620 and 0.586, respectively, yielding a difference of 0.034 ( ≥ 0.01). The DeLong test showed no statistically significant difference between the two (*Z* = 1.496, *P* = 0.134), and Rule 1 failed to identify a unique candidate. According to Rule 2, SIRI and PIV demonstrated comparable breadth of external clinical validation; Subsequently, based on Rule 3, SIRI (composed of Neutrophils, Lymphocyte, and Platelet—three variables) was retained over PIV (requiring four variables) due to greater simplicity.

Ultimately, five candidate indicators (GLR, SIRI, GPR, NPR, MHR) were retained. Each was combined with the Baseline model to construct five competing models.

### 4.5 Model comparison and evaluation

Six variables—mean platelet volume, blood glucose, D-dimer, history of alcohol consumption, leukoaraiosis, and pulmonary infection—were incorporated into binary logistic regression to construct the baseline model. Subsequently, five composite biomarkers were sequentially added to evaluate changes in predictive efficacy across models.

Regarding discrimination ability, incorporating GLR yielded a marginally significant increase in the baseline model’s AUC (from 0.852 to 0.882; ΔAUC = 0.030, *P* = 0.057) (Table 6). After Bootstrap correction, GLR maintained the highest corrected AUC (0.861) (Table 7). The ROC curve demonstrated that the GLR curve consistently occupied the highest position, indicating the best predictive capability (Figure 4).

**TABLE 6:** Comparison of ROC curve AUC for predicting hemorrhagic transformation between the baseline model and the baseline model combined with various inflammatory composite biomarkers.

Model	AUC	95% CI	Δ AUC	*z*	*p*
Base	0.852	0.785–0.907			
Base +GLR	0.882	0.827–0.930	0.03	−1.903	0.057
Base +NPR	0.863	0.802–0.914	0.01	−1.575	0.115
Base +MHR	0.857	0.794–0.912	0.005	−0.535	0.593
Base +SIRI	0.856	0.793–0.915	0.004	−1.139	0.255
Base +GPR	0.852	0.785–0.909	−0.001	0.277	0.782

**TABLE 7:** AUC optimism correction results for each model based on bootstrap resampling (1,000 iterations).

Index	Original AUC	Bootstrap AUC (mean)	Optimism	Corrected AUC
Base	0.852	0.863	0.018	0.835
Base +GLR	0.882	0.891	0.021	0.861
Base +NPR	0.863	0.881	0.023	0.84
Base +MHR	0.857	0.87	0.022	0.835
Base +SIRI	0.856	0.872	0.021	0.835
Base +GPR	0.852	0.867	0.022	0.829

**FIGURE 4:**
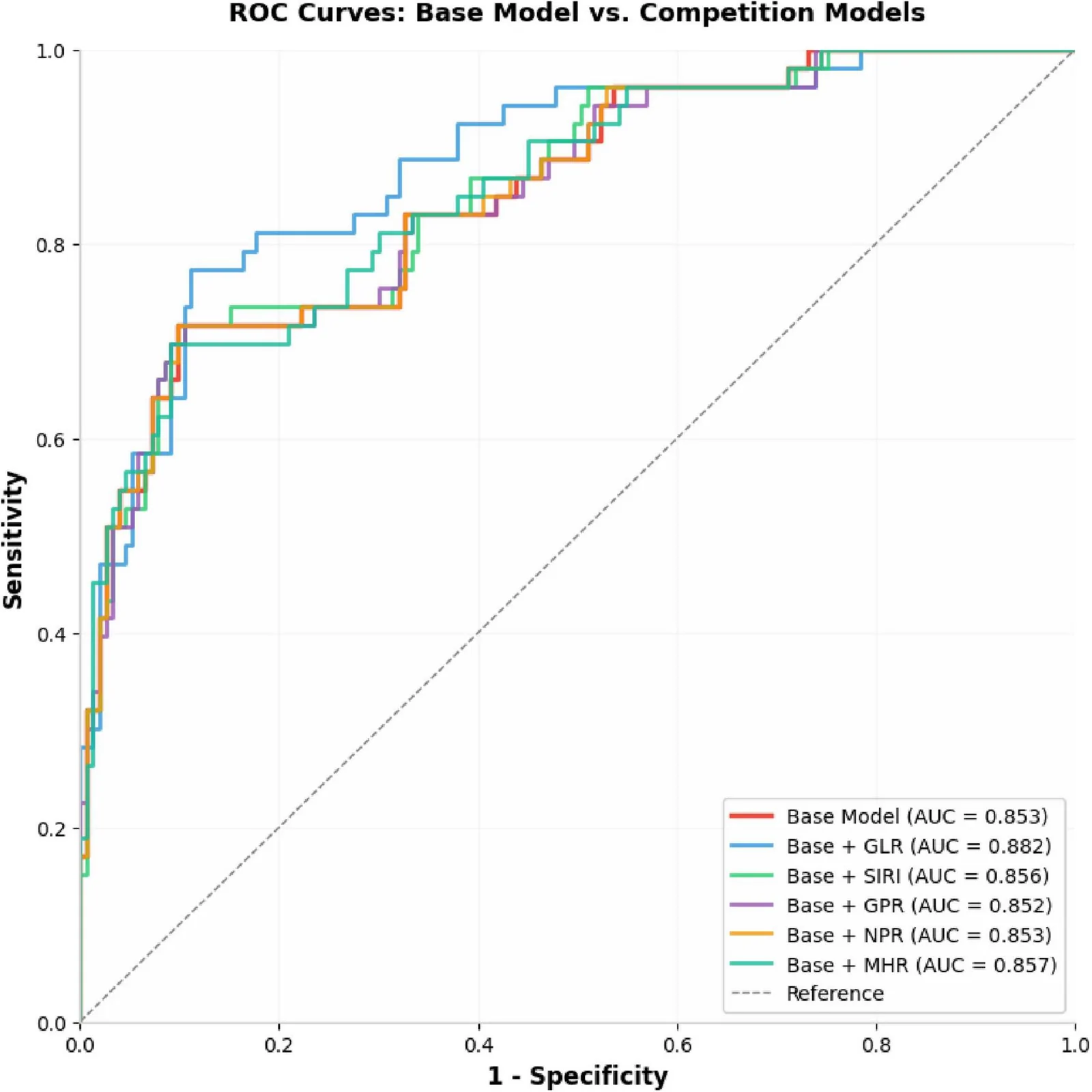
ROC curves of the baseline model and the five composite biomarker-enhanced models for predicting hemorrhagic transformation.

Decision curve analysis (DCA) revealed significant overlap between the curves for GLR, the baseline model, and other composite biomarker models, with net benefit differences < 0.03. This suggests that relying solely on GLR to guide hemorrhage prevention strategy has limited clinical value; it requires integration with multidimensional factors such as imaging and procedural information for comprehensive assessment (Figure 5).

**FIGURE 5:**
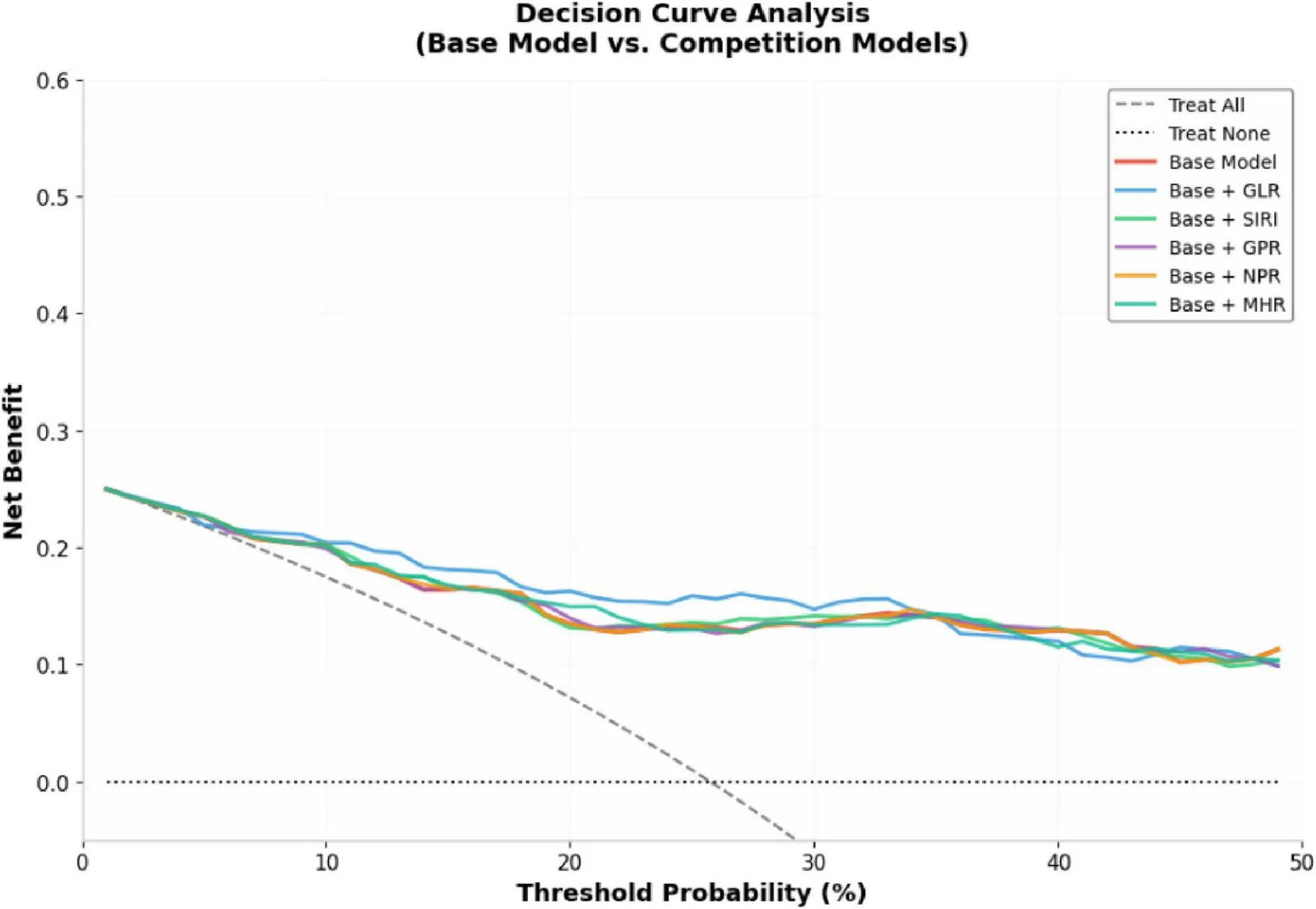
Decision curve analysis comparing net benefit across models at different threshold probabilities.

Concerning calibration, the baseline model incorporating GLR showed the most substantial improvement. While MHR demonstrated superior model fit, its improvement in probabilistic accuracy remains modest. Conversely, GPR was the sole biomarker exhibiting no improvement in calibration (Figure 6 and Table 8).

**FIGURE 6:**
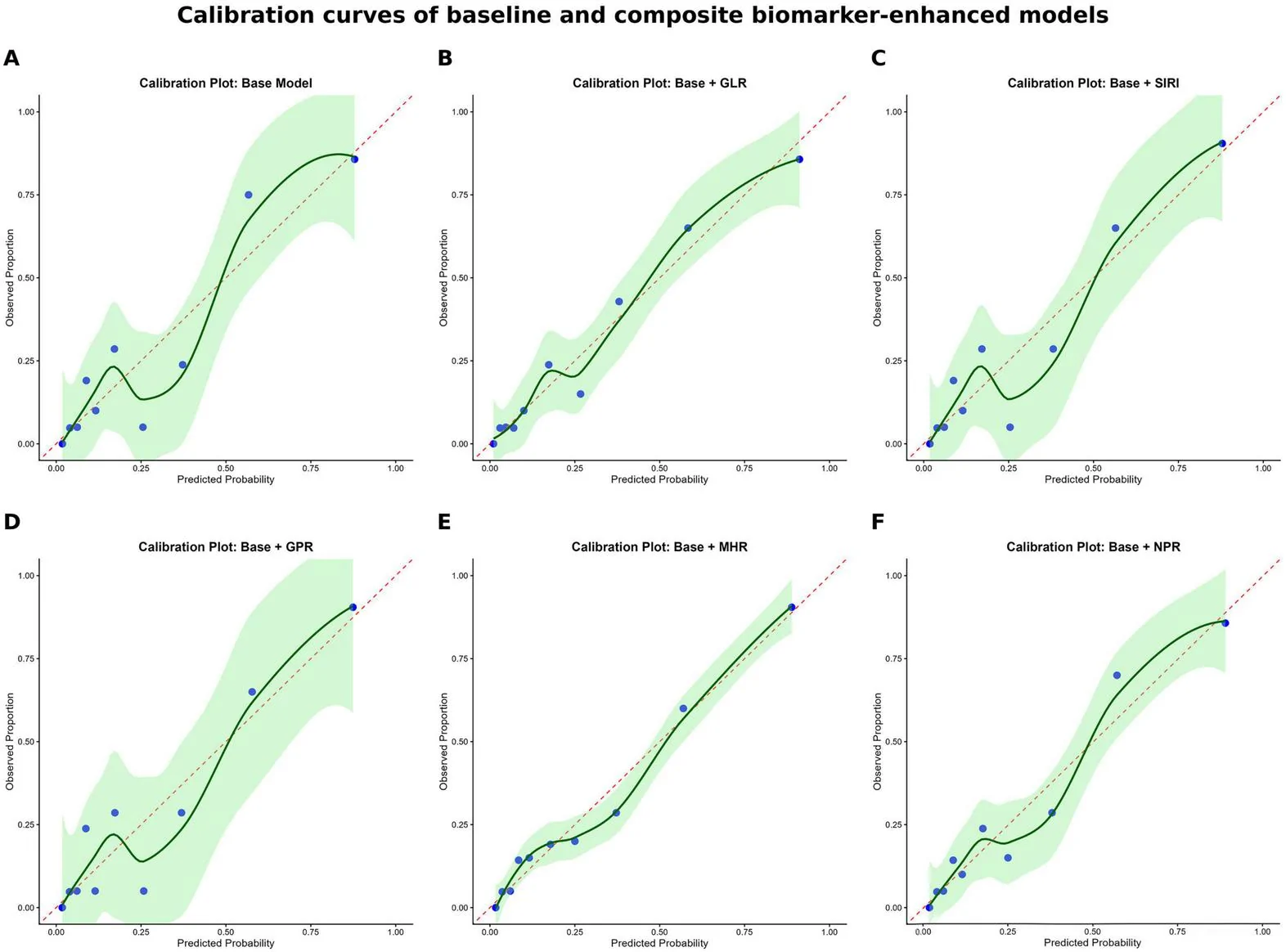
Calibration of the baseline and composite biomarker-enhanced models. Observed versus predicted probability of hemorrhagic transformation is shown for **(A)** base, **(B)** base + GLR, **(C)** base + SIRI, **(D)** base + GPR, **(E)** base + MHR and **(F)** base + NPR; the green solid line is the apparent calibration curve and the dashed diagonal ideal calibration. Calibration improved most after adding GLR (Hosmer-Lemeshow *P* = 0.858 vs. 0.081 for base), whereas GPR showed no improvement; full Hosmer-Lemeshow and Brier score results are given in Table 8.

**TABLE 8:** Comparison of brier scores and Hosmer-Lemeshow goodness-of-fit tests across predictive models.

Index	HL χ ^2^	df	HL *P*-value	Brier score	Change vs. base
Base	14.03	8	0.081	0.118	–
GLR	3.99	8	0.858	0.1116	↓5.4%
MHR	2.73	8	0.950	0.1164	↓1.4%
NPR	5.29	8	0.726	0.1168	↓1.1%
SIRI	11.16	8	0.193	0.1179	↓0.1%
GPR	14.89	8	0.061	0.1182	↑0.1%

Regarding net reclassification improvement, GLR was the only biomarker among the five that achieved statistical significance. SIRI produced a negative net reclassification improvement in the event group (-0.36), suggesting a potential risk of missed diagnosis. Neither MHR nor NPR achieved significant Integrated Discrimination Improvement in the test set. Both exhibited significant directional limitations, restricting their clinical applicability. MHR’s strength is primarily in identifying high-risk hemorrhage patients; however, its ability to exclude low-risk patients is weak, potentially leading to overtreatment. In contrast, NPR helps rule out non-hemorrhagic patients but carries the risk of misclassifying hemorrhagic patients as low-risk, raising concerns about missed detection (Table 9).

**TABLE 9:** Comparison of integrated discrimination improvement (IDI) and net reclassification improvement (NRI) between the baseline model and the five composite biomarker-enhanced models.

Model	IDI	95% CI	Continuous NRI	NRI Event	NRI NonEvent
Base + GLR	0.0376	0.002 to 0.078	0.712	0.170	0.542
Base + SIRI	0.0014	−0.005 to 0.008	0.315	−0.358	0.673
Base + GPR	0.0001	−0.004 to 0.004	0.158	−0.019	0.176
Base + NPR	0.008	−0.005 to 0.022	0.484	−0.019	0.503
Base + MHR	0.009	−0.007 to 0.024	0.406	0.321	0.085

Among the five composite biomarkers, predictive efficacy ranks as follows: GLR > NPR ≈ MHR ≈ SIRI > GPR. GLR is the only biomarker that marginally improves the baseline model’s predictive efficacy, demonstrating optimal performance in discrimination ability, calibration, and net reclassification improvement. NPR, MHR, and SIRI provide limited gain, failing to substantially enhance overall predictive efficacy compared to the baseline model. GPR not only failed to improve model performance but also showed no improvement in calibration, yielding no clinical net benefit.

### 4.6 Supplementary experiments: sensitivity analysis regarding collinearity between blood glucose and GLR

To rigorously rule out the possibility that the incremental value of GLR was merely an artifact of blood glucose redundancy, we conducted three sensitivity analyses (see Supplementary Figure S1 and Supplementary Table S1).

When blood glucose was excluded from the baseline model, discrimination dropped substantially, with the AUC decreasing from 0.852 to 0.790. However, adding GLR to this adjusted baseline model raised the AUC to 0.865 (ΔAUC = +0.075, DeLong test *P* = 0.003; bootstrap-corrected AUC = 0.853; Brier score = 0.117; Hosmer–Lemeshow test *P* = 0.656), demonstrating that GLR provides robust incremental predictive value independent of blood glucose redundancy.

In the original baseline model (which retained blood glucose), substituting GLR with “1/lymphocyte count” yielded an AUC of 0.882, numerically identical to that of the original “baseline + GLR” model (Brier score = 0.111; Hosmer–Lemeshow test *P* = 0.955). This indicates that, as a composite ratio, GLR’s integration of metabolic and immune information is statistically equivalent to including its constituent variables separately in the model.

Finally, linear regression analysis revealed that blood glucose explained 37.1% of the variance in GLR (*R*^2^ = 0.371). After removing this shared variance, incorporating the orthogonalized GLR residuals into the baseline model still achieved an AUC of 0.882 (Brier score = 0.112; Hosmer–Lemeshow test *P* = 0.858; bootstrap-corrected AUC = 0.871), showing no loss in predictive performance compared to the original “baseline + GLR” model.

In summary, these results demonstrate that the incremental predictive value of GLR reflects genuine metabolic–immune integration, rather than a mathematical artifact arising from collinearity with blood glucose.

## 5 Discussion

This retrospective study analyzed 206 acute anterior circulation LVO stroke patients treated with mechanical thrombectomy. Hemorrhagic transformation occurred in 53 cases (25.7%). Multimarker stratification revealed GLR’s incremental explanatory power for HT risk, encapsulating both metabolic stress burden and immune/inflammatory dysregulation metrics.

The GLR captures two distinct pathophysiological pathways by integrating hyperglycemic injury stress and low immune buffering capacity.

Elevated admission or acute-phase blood glucose constitutes an independent risk factor for hemorrhagic transformation following ischemic stroke. It promotes hemorrhagic transformation by activating the oxidative stress (ROS)-matrix metalloproteinase (MMP) cascade reaction, thereby compromising BBB integrity.

Hyperglycemia combined with cerebral ischemia/reperfusion significantly accelerates reactive oxygen species generation, subsequently upregulating MMP-2 and MMP-9 activity. Among them, MMP-2 directly mediates extracellular degradation of occludin, while caveolin-1 mediates intracellular redistribution of claudin-5. Both have been identified as key molecular events in early blood-brain barrier disruption (Liu et al., 2012). Meanwhile, MMP-9 degrades type IV collagen and laminin, impairing microvascular structural integrity (Gu et al., 2005; Kamada et al., 2007; Romanic et al., 1998; Rosell et al., 2008). Clinical specimen studies demonstrate that MMP-9 levels in hemorrhagic lesion microvascular endothelium strongly correlate with the degree of blood-brain barrier rupture (Rosell et al., 2008; Romanic et al., 1998; Zhang et al., 2018). Furthermore, hyperglycemia contributes to reduced zonula occludens-1 (ZO-1) through MMP-dependent extracellular degradation, caveolin-1-mediated intracellular translocation, and autophagy-lysosomal pathways, further increasing blood-brain barrier permeability (Romanic et al., 1998).

Conversely, hyperglycemia activates pro-inflammatory transcriptional programs including NF-κB/AP-1/Egr-1, upregulating adhesion molecule expression and intensifying leukocyte-endothelial interactions. Simultaneously, it alters local hemostatic balance through tissue factor (TF) and plasminogen activator inhibitor-1 (PAI-1), creating a pro-thrombotic and pro-inflammatory microenvironment (Salman et al., 2022; Jiang et al., 2020; Jickling et al., 2014; Lakhan et al., 2013).

Post-stroke lymphocytopenia stems from HPA axis-sympathetic activation-driven cortisol/catecholamine-dependent lymphocyte redistribution and consumption, clinically manifesting as reduced absolute lymphocyte counts (Courties et al., 2019). This study demonstrated significantly lower lymphocyte counts in the HT group compared to the non-HT group.

The significance of lymphocytopenia extends beyond abnormalities in inflammatory biomarkers. Salas-Perdomo et al. (2018a) demonstrated through animal models that functional P-selectin binding sites are crucial for T cell-mediated hemostatic effects, indicating that T cells interact with platelets via P-selectin glycoprotein ligand-1 (PSGL-1), participating in “immune-hemostasis” coupling (Salas-Perdomo et al., 2018b). Consequently, in the MT scenario, lymphocytopenia exacerbates neutrophil-driven inflammatory injury on one hand and impairs the hemostatic protection mediated by T cell binding to platelets through PSGL-1/P-selectin interactions on the other, thereby increasing HT risk (Salas-Perdomo et al., 2018a).

The NLR, SII, PLR and other metrics reflect an imbalance between neutrophil/platelet and lymphocyte counts. Although these metrics have been confirmed as correlating with HT (Hao et al., 2022; Pikija et al., 2018) and stroke outcomes (Gencdal et al., 2022; Hou et al., 2021; Hu et al., 2023; Yilmaz and Sönmezler, 2026; Zhou et al., 2025) after mechanical thrombectomy, they fundamentally serve as surrogate markers for the inflammatory burden (Agard et al., 2024), with their prognostic value confined to inflammatory outcomes (Wang et al., 2023; Yuan et al., 2025). In the present study, covariates such as pulmonary infections were already incorporated into the model, making the marginal information contributed by pure inflammatory ratios approach saturation. The SII exhibited negative net reclassification in this study, further confirming the limitation of relying solely on the inflammatory dimension.

SIRI and PIV attempt to capture broader inflammatory signaling through multi-cell type integration, but remain fundamentally a weighted expression of inflammatory burden. PIV incorporates four blood cell variables, introducing high collinearity risk and impairing clinical interpretability; although SIRI calculation is relatively concise, it also fails to provide stable incremental value in model competition due to the absence of a metabolic dimension.

GPR shows association with HT following AIS (Alamri et al., 2024). The univariate discriminatory direction of GPR in this study aligns with this finding, but calibration showed no improvement when incorporated into a multivariate model adjusting for covariates including infections and leukoaraiosis. Hypokalemia can arise from factors such as systemic sympathetic activation and β^2^ receptor-mediated potassium influx, which correlate with stroke severity yet lack a direct causal link to hemorrhagic transformation. Therefore, the predictive efficacy of GPR may partially originate from confounding pathways rather than directly reflecting HT pathology. When covariates more proximate to the causative pathways were incorporated into the model, the independent contribution of GPR became attenuated.

While NPR and MHR aim to integrate distinct biological dimensions, both biomarkers fail to comprehensively cover metabolic and immune aspects. NPR reflects the interplay between neutrophil-driven inflammation and platelet-mediated hemostasis. MHR links monocyte-related inflammation with lipid metabolism. However, HDL-C often exhibits reactive fluctuations during acute stroke stress and demonstrates weak direct evidence for its association with HT. In this study, each biomarker exhibits directional limitations: MHR effectively identifies patients at high bleeding risk but possesses limited ability to exclude low-risk patients, potentially resulting in overtreatment; NPR excels at excluding patients without hemorrhage but risks misclassifying hemorrhagic patients as low-risk, creating potential missed diagnoses.

This study demonstrates that GLR, a composite biomarker derived from routine laboratory tests, provides incremental predictive information to traditional clinical-laboratory models. This finding is consistent with evidence supporting GLR as a “metabolic-immune integrative factor” in severe cerebrovascular disease cohorts. Li et al. (2025) confirmed in their 2025 large-sample study that GLR provides independent prognostic value for all-cause mortality and consciousness disorders in patients with severe cerebrovascular disease, adding incremental discrimination ability to traditional scoring systems. These results corroborate such findings and support the practical value of GLR in predicting adverse stroke outcomes.

HT occurrence is jointly determined by recanalization quality, infarct core and collateral status, microbleeds/leukoaraiosis burden, perioperative blood pressure management, and antithrombotic strategy (Ciacciarelli et al., 2023; Goyal et al., 2016). This study’s decision curve analysis indicates limited net benefit difference between GLR and the baseline model, suggesting GLR alone is insufficient to drive hemorrhage prevention decisions. Integration with medical imaging and intraoperative information is required—GLR is better suited as an “early warning layer” for bedside risk stratification rather than replacing medical imaging and intraoperative decision-making tools. Notably, both glucose and lymphocyte counts are routinely available within 2 h of admission in most centers, making GLR a readily accessible and cost-effective tool for early perioperative risk assessment.

## 6 Study limitations and future directions

This study has several limitations. First, the single-center retrospective design may introduce selection bias, and conclusions have limited external validity. Second, blood glucose and lymphocyte measurements relied solely on single admission readings, failing to capture dynamic trajectories during the acute phase—particularly peri-reperfusion—and unable to reflect the cumulative or time-varying effects of blood glucose fluctuations and lymphocyte temporal changes on HT risk. Third, the sample size limited the statistical power of stratified analyses based on key variables including recanalization time window, collateral status, and history of diabetes. Fourth, procedure-related quality indicators were not consistently documented in the electronic medical records and were therefore not included in the analysis. Fifth, composite biomarkers were calculated from raw laboratory values without log-transformation; given their skewed distributions, this may violate the linearity assumption of logistic regression, though the internal validation results suggest acceptable model performance despite this limitation.

Future research may advance in the following directions: (1) Conduct longitudinal acquisition of GLR temporal data at admission, immediately post-procedure, 24 h, and 72 h to construct trajectory models for assessing time-dependent predictive value and identifying optimal risk stratification windows; (2) In multicenter cohorts, utilize uniform scanning protocols and revascularization assessment criteria to validate the robustness and generalizability of GLR across varying thrombectomy devices and postoperative antithrombotic strategies.

## 7 Conclusion

Among nine blood composite biomarkers, the Glucose-to-Lymphocyte Ratio (GLR) provides the greatest incremental predictive value for hemorrhagic transformation following mechanical thrombectomy in acute anterior circulation LVO stroke patients. As a routinely accessible metabolic-immune integrative index derived from admission laboratory tests, GLR may serve as an early warning layer for perioperative risk stratification. However, integration with neuroimaging and procedural information remains essential for comprehensive clinical decision-making.

## Data Availability

The original contributions presented in the study are included in the article/Supplementary material, further inquiries can be directed to the corresponding author.
