# Supplementary material for: Predictive value of blood composite biomarkers for hemorrhagic transformation following mechanical thrombectomy in acute anterior circulation large vessel occlusion stroke patients
Source: Front Neurosci. 2026 Sep 14;20:1907532. doi: 10.3389/fnins.2026.1907532 (PMC13616940; doi:10.3389/fnins.2026.1907532)
Supplement: Supplementary file 1 [file Supplementary_file_1.docx]

| Model | AUC | 95% CI | ΔAUC | DeLong P* | Brier | H-L P | Bootstrap AUC |
| --- | --- | --- | --- | --- | --- | --- | --- |
| Base (Original) | 0.852 | 0.784~0.920 |  |  | 0.118 | 0.081 | 0.838 |
| Base+GLR(Original) | 0.882 | 0.820~0.944 | 0.03 | 0.057 | 0.1116 | 0.858 | 0.871 |
| Base (no glucose) | 0.79 | 0.713~0.868 |  |  | 0.1404 | 0.729 | 0.779 |
| Base(noglucose)+GLR | 0.865 | 0.799~0.930 | +0.075 | 0.003 | 0.1169 | 0.656 | 0.853 |
| Base+1/lymphocyte | 0.882 | 0.820~0.944 | 0.03 | 0.06 | 0.1113 | 0.955 | 0.868 |
| Base+GLR residualized | 0.882 | 0.820~0.944 | 0.03 | 0.057 | 0.1116 | 0.858 | 0.871 |

*DeLong test P values: Base + GLR, Base + 1/lymphocyte, and Base + GLR residualized were compared against Base (Original); Base (no glucose) + GLR was compared against Base (no glucose). Bootstrap optimism correction was performed with 1000 iterations.

Table S1 Sensitivity analysis of the incremental predictive value of GLR independent of blood glucose.

Figure S1. Sensitivity analysis regarding the collinearity between blood glucose and GLR.

(A) Original models: baseline model and baseline model + GLR.

(B) Strategy A: Adding GLR improved discrimination after excluding blood glucose from the baseline model.
